# Single-Step Genomic Reaction Norm and ssGWAS Reveal Heat-Load-Dependent Genetic Architecture of Age at Puberty in Yorkshire Gilts

**DOI:** 10.3390/ani16193085

**Published:** 2026-09-30

**Authors:** Chong Zhang, Jinglei Si, Xiaoxiao Song, Youen Xiao, Wenzhou Wang, Borong Liang, Shaoyun Li, Peiqing Cong, Yaosheng Chen, Xiaohong Liu

**Affiliations:** 1 State Key Laboratory of Biocontrol, School of Life Sciences, Sun Yat-sen University, Guangzhou 510275, China; zhangchongzczc@sina.com (C.Z.); songxx9@mail2.sysu.edu.cn (X.S.); congpq@mail.sysu.edu.cn (P.C.); chyaosh@mail.sysu.edu.cn (Y.C.); 2 Guangxi State Farms Yongxin Animal Husbandry Group Co., Ltd., Nanning 530022, China; jinglei7139@126.com (J.S.); hxxye2018@163.com (Y.X.); 3 Guangdong Guangken Animal Husbandry Group Co., Ltd., Guangzhou 510612, China; wangwenzhou7@163.com (W.W.); brliang@scau.edu.cn (B.L.); lishaoyun91@163.com (S.L.)

**Keywords:** heat stress, genotype-by-environment interaction, reaction norm model, genomic estimated breeding value, environmental sensitivity, reproductive resilience

## Abstract

Heat stress adversely affects porcine reproduction, but genetic variation in the response of age at puberty (AP) to heat exposure remains poorly characterized in gilts. We analyzed 4438 AP records from Yorkshire gilts using adjusted meteorological data and compared three heat-exposure gradients across five prepubertal windows. Pedigree- and single-step genomic reaction-norm models were fitted, followed by single-step genome-wide association analyses of the intercept and slope. Among the 15 exposure scenarios, the best-fitting model used the proportion of sustained heat-stress days in the 14-day window before first observed estrus; a one-standard-deviation increase in this measure was associated with a 7.64-day increase in AP. Both reaction-norm models detected significant genetic variance for environmental sensitivity (*p* < 0.001). The genomic intercept–slope genetic correlation was −0.524, and genetic variances and correlations varied across the exposure gradient. GEBVs rankings at the 10th and 90th percentiles of the exposure gradient were negatively correlated (Spearman’s ρ = −0.742), and the most favorable 10% of gilts did not overlap between these two exposure levels. Intercept and slope window contributions were strongly correlated (Spearman’s ρ = 0.822), although component-specific regions were also detected. Functional annotation highlighted candidate genes and biological processes related to neuroendocrine regulation, ovarian development, mitochondrial and redox homeostasis, and cellular stress responses. These findings reveal genetically heterogeneous heat sensitivity in AP, supporting environment-specific genomic evaluation for reproductive robustness in gilts.

## Simple Summary

Heat stress can delay puberty in gilts, increasing nonproductive days and reducing reproductive efficiency. However, gilts may differ genetically in how their puberty timing responds to sustained heat exposure. We analyzed 4438 age-at-puberty records from Yorkshire gilts. A higher proportion of sustained heat-stress days during the preceding 14 days was associated with later puberty. Genomic reaction-norm analysis showed that gilts differed genetically in heat sensitivity and that their genetic rankings changed markedly between low- and high-exposure conditions. Gilts ranked favorably in one environment were therefore not necessarily favorable in the other. Genome-wide analysis further identified the shared and component-specific genomic regions associated with average puberty timing and heat responsiveness. These findings indicate that heat responsiveness of age at puberty can be considered a reproductive-efficiency and resilience phenotype. After external validation, environment-specific genetic evaluations could help breeders select gilts that combine earlier puberty with greater stability under heat stress.

## 1. Introduction

Climate warming and increasingly frequent periods of elevated temperature pose growing challenges to efficiency, the welfare of domestic animals or pigs, and the sustainability of livestock production [1]. This challenge is particularly relevant to commercial pig production in hot-humid southern China, where prolonged periods of high temperature and humidity frequently coincide with gilt development. Pigs are particularly vulnerable to high ambient temperature because of their limited capacity for evaporative heat loss, and thermal stress can disrupt feed intake, metabolism, reproductive function, and production efficiency [2,3]. Within maternal pig populations, the successful development of replacement gilts is fundamental to breeding-herd productivity. Age at puberty (AP), commonly defined as age at first observed standing estrus, determines when a gilt becomes reproductively competent and influences the duration of nonproductive rearing. Earlier puberty is generally associated with fewer nonproductive days and improved retention in the breeding herd, although reported relationships between AP and lifetime litter output are not entirely consistent [4,5]. AP is also moderately heritable, and previous physiological and genomic studies have implicated neuroendocrine, metabolic, and ovarian processes in its regulation [5,6,7,8]. However, most genetic studies of AP have estimated effects averaged across environments and have not examined whether the genetic expression of this trait changes with thermal exposure.

Evidence that elevated temperature can disrupt gilt reproductive development has accumulated from both controlled experiments and commercial populations. In a controlled study of 40 prepubertal gilts, chronic exposure to 33.3 °C substantially reduced the proportion attaining puberty by 230 days of age and delayed average AP [9]. Recent studies have used temperature–humidity index (THI) to identify levels of heat exposure associated with physiological and reproductive changes in pigs. De Prekel et al. analyzed approximately 9000 individual records and identified 71 ≤ THI ≤ 76 as an early-warning range based on changes in respiration rate and rectal temperature [10]. In sows, exposure to approximately THI 72–78 has been associated with increased oxidative damage to lipids, proteins, and DNA together with impaired reproductive performance [11]. More recently, Chavez-Flores et al. reported that reproductive outcomes were generally more favorable below a THI of approximately 74 [12]. Collectively, these findings indicate that thermal responses in pigs can emerge around THI 72–74, although the precise threshold varies with the age, sex, and production stage.

Experimental studies also provide a biological basis for the association between heat exposure and delayed reproductive maturation. Elevated temperature suppressed follicle-stimulating hormone and luteinizing hormone secretion and impaired follicular growth in prepubertal gilts [13]. At the population level, an analysis of 37,362 gilts from 101 commercial herds associated a greater number of hot days during development with later age at first mating [14]. Thermal exposure during specific periods around insemination has also been associated with reduced farrowing rate and number of total born piglets, with stronger relationships in gilts than in multiparous sows [15]. More recently, cyclic heat stress was shown to alter ovarian proteins involved in metabolism, protein processing, immune activation, and gene regulation in prepubertal gilts [16]. However, identifying a biologically relevant heat-sensitive period remains challenging because thermal responses occur over different temporal scales. Acute physiological responses, such as increased respiration rate and rectal temperature, may occur rapidly after thermal challenge, whereas reproductive outcomes may reflect cumulative exposure over several days or weeks. Moreover, heat exposure has been defined relative to different physiological or production stages across studies, making it difficult to establish a standardized exposure window.

The reproductive consequences of heat exposure may also differ genetically among animals. Genotype-by-environment interaction occurs when genetic effects on a trait differ across environmental conditions and may alter genetic variance and cross-environment genetic correlations. Reaction-norm models provide a framework for characterizing this interaction by describing each animal’s genetic merit as a function of a continuous environmental gradient [17]. In a linear reaction norm, the intercept represents an animal’s genetic merit for AP at the average level of heat exposure, whereas the slope describes how its genetic merit changes as heat exposure increases. A larger positive slope therefore indicates greater sensitivity to increasing heat load, whereas a flatter slope indicates greater stability across the environmental gradient [18]. Genomic reaction-norm studies in maternal pig populations have demonstrated heritable heat sensitivity for number of total born piglets, number born alive, piglet birth weight, farrowing interval, and other reproductive traits [19,20,21]. Despite these advances, genetic heterogeneity in the association between heat exposure and gilt AP remains largely unexplored. It is also unknown whether genetic variation in AP at an average exposure level and variation in its sensitivity to increasing heat exposure arise predominantly from shared genomic regions or from component-specific mechanisms.

Therefore, this study used records from commercial Yorkshire gilts raised on two farms in hot-humid southern China to (1) compare alternative heat-exposure descriptors and identify the environmental gradient most strongly associated with AP; (2) quantify genetic variation in AP and its sensitivity to increasing heat exposure using genomic reaction-norm models; and (3) characterize and compare genomic regions contributing to baseline AP and environmental sensitivity using ssGWAS. By quantifying environment-specific genetic merit, this framework may provide a basis for identifying gilts with more stable reproductive performance under sustained heat load and for incorporating reproductive heat resilience into breeding programs in hot-humid production systems.

## 2. Materials and Methods

### 2.1. Ethics Approval and Consent to Participate

Ethical approval and consent to participate were not applicable. This study used only retrospective production records collected during routine commercial farm management. No animals were handled and no biological samples were collected specifically for this study.

### 2.2. Phenotypic Data and Definition of Age at Puberty

Phenotypic records were obtained from Yorkshire gilts raised in two commercial pig farms located in Zhanjiang, Guangdong Province, and Nanning, Guangxi Province, China. Age at puberty (AP) was calculated as the interval, in days, between the date of birth and the date of first observed estrus. After quality control, 4438 AP records were retained for downstream analyses.

### 2.3. Genotype and Pedigree Data

Genotype data were available for 5753 Yorkshire animals and were generated using the Zhongxin I 50 K Porcine Breeding Chip (Beijing Compass Agritechnology Co., Ltd., Beijing, China). SNP coordinates were assigned according to the Sscrofa11.1 reference genome assembly. Marker-level preprocessing excluded variants located on the sex chromosomes, variants without assigned genomic coordinates, duplicated markers, and variants with nonstandard or non-biallelic alleles, leaving 35,712 autosomal biallelic SNPs for subsequent quality control. Basic quality control was performed using PLINK v2.0 (https://www.cog-genomics.org/plink/2.0/, accessed on 28 September 2026) [22]. Individuals with more than 10% missing genotypes were excluded, and SNPs with more than 5% missing genotypes or a minor allele frequency (MAF) below 0.05 were removed (--mind 0.1 --geno 0.05 --maf 0.05). These thresholds corresponded to minimum call rates of 90% for individuals and 95% for SNPs. Two animals were excluded because their genotype missingness exceeded 10%. At the marker level, 299 SNPs were removed because of genotype missingness >5%, and a further 7435 SNPs were removed because their MAF was <0.05. After quality control, 5751 animals and 27,978 autosomal SNPs remained. Of these animals, 4438 gilts had AP records.

Pedigree information was obtained from the breeding records of the two commercial farms. The pedigree included 8496 animals and was traced back for up to three ancestral generations from the gilts. The pedigree-based numerator relationship ***A*** matrix was constructed using the complete pedigree, thereby incorporating relationships between gilts and their recorded ancestors.

### 2.4. Meteorological Data and Adjustment of Barn Thermal Conditions

Historical meteorological data from 2020 to 2024 were collected for the two farm locations in Zhanjiang and Nanning. Hourly outdoor air temperature and relative humidity were extracted from historical datasets provided by the European Centre for Medium-Range Weather Forecasts (ECMWF) and the National Aeronautics and Space Administration (NASA) [23] and were used to calculate thermal-stress indicators.

Because gilts were housed in mechanically ventilated barns equipped with Cool Cells and basket fans (Munters Group AB, Kista, Sweden), outdoor meteorological records were adjusted to approximate indoor thermal conditions. To establish the relationship between outdoor meteorological conditions and the thermal environment inside the barns, on-farm temperature and relative humidity were monitored for one month using integrated temperature–humidity meters. Measurements were taken at four fixed times each day (07:30, 11:00, 14:00, and 17:00), covering key periods before and after feeding. Within each barn, measurements were obtained at three spatial locations (front, middle, and rear) at approximately 1.5 m above the floor and away from direct airflow from the basket fans. The three measurements were averaged at each sampling time to represent the barn-level thermal condition. Measurements were conducted under routine farm-management conditions, with the ventilation and cooling systems operated according to the standard farm protocol. Comparison of the within-barn measurements with the corresponding outdoor meteorological records indicated a consistent relationship between outdoor and indoor thermal conditions and provided the empirical basis for the cooling adjustments described below.

According to farm management protocols, when outdoor temperature was 25 °C or lower, only basket fans were operated, whereas Cool Cells were not activated. Under this condition, no cooling adjustment was applied, and outdoor temperature was used to approximate barn temperature. When outdoor temperature exceeded 25 °C, both Cool Cells and basket fans were activated. Based on on-farm measurements during Cool Cell operation, the difference between outdoor and indoor temperature depended on relative humidity. Specifically, indoor temperature was reduced by 10 °C when relative humidity was below 40%, by 6 °C when relative humidity ranged from 40% to 60%, and by 3 °C when relative humidity exceeded 60%. The adjusted barn temperature was therefore calculated as

(1) $$T_{\mathrm{adj},h}=T_{\mathrm{out},h}-C_{h},$$

where $T_{\mathrm{adj},h}$ is the adjusted barn temperature at hour (h), $T_{\mathrm{out},h}$ is the corresponding outdoor temperature, and $C_{h}$ is the cooling adjustment. When $T_{\mathrm{out},h}\;$ was 25 °C or lower, $C_{h}\;$ was set to 0 °C. When $T_{\mathrm{out},h}$ exceeded 25 °C, $C_{h}\;$ was set to 10 °C at relative humidity below 40%, 6 °C at relative humidity between 40% and 60%, and 3 °C at relative humidity above 60%.

Hourly adjusted temperature–humidity index (THI) was calculated using the adjusted barn temperature and the corresponding hourly relative humidity as follows [24]:

(2) $$THI_{\left(adj,h\right)}=\left(1.8T_{\left(adj,h\right)}+32\right)-\left(0.55-0.0055RH_{h}\right)\left(1.8T_{\left(adj,h\right)}-26\right),$$

where $THI_{\left(adj,h\right)}$ is the adjusted THI at hour (h), $T_{\left(adj,h\right)\;}\;$ is the adjusted barn temperature in degrees Celsius, and $RH_{h}$ is the corresponding relative humidity expressed as a percentage. A day was classified as a sustained heat-stress day when ${\mathrm{THI}}_{\mathrm{adj},h}$ exceeded 72 for at least six consecutive hours. THI 72 was selected as a conservative operational cutoff based on previous swine studies reporting physiological and reproductive responses beginning around THI 71–74 [10,11,12], while the six-hour criterion was used to identify sustained rather than transient exposure.

### 2.5. Genetic Parameter Estimation and Reaction Norm Analysis

The selected environmental gradient, propEG14, was standardized before model fitting as follows:

(3) $$propEG14_{z}=\frac{\left(propEG14-mean\left(propEG14\right)\right)}{SD\left(propEG14\right)},$$

where propEG14 represents the proportion of sustained heat-stress days during the 14 days preceding the first observed estrus.

Variance components for AP were first estimated using the following pedigree-based single-trait animal model:

(4) y=Xb+Za+e,

where ***y*** is the vector of AP records, ***b*** is the vector of fixed effects, including farm and year of first observed estrus, ***a*** is the vector of additive genetic effects, and ***e*** is the vector of residuals. ***X*** and ***Z*** are the corresponding incidence matrices. Additive genetic relationships among animals were described using the pedigree-based relationship ***A*** matrix. The additive genetic effects were assumed to follow a ~  N(0, ***A*** $\sigma_{a}^{2}$), where ***A*** is the pedigree-based numerator relationship matrix and $\sigma_{a}^{2}$ is the additive genetic variance. Residuals were assumed to follow ***e*** ~  N(0, *I* $\sigma_{e}^{2}$), where ***I*** is an identity matrix and $\sigma_{e}^{2}$ is the residual variance.

To estimate the population-level association between sustained heat-stress exposure and AP, propEG14_z was included as a fixed covariate in a separate animal model:

(5) y=Xb+qβ+Za+e,

where ***q*** is the vector of propEG14_z values and β is the fixed regression coefficient representing the average change in AP associated with a one-standard-deviation increase in sustained heat-stress exposure.

Heritability was calculated as $h^{2}=\frac{\sigma_{a}^{2}}{{(\sigma }_{a}^{2}+\sigma_{e}^{2})}$, where $\sigma_{a}^{2}$ and $\sigma_{e}^{2}$ are the additive genetic and residual variances, respectively.

Genetic variation in AP responsiveness to sustained heat-stress exposure was subsequently evaluated using the following linear reaction norm model [25,26,27]:

(6) $$y_{i}=X_{i}b+a_{0i}+q_{i}a_{1i}+e_{i},$$

where $y_{i}$ is the AP record of gilt (i), $X_{i}b$ represents the fixed effects, $q_{i}$ is the standardized propEG14 value, $a_{0i}$ is the animal-specific additive genetic intercept, $a_{1i}$ is the animal-specific additive genetic slope, and $e_{i}$ is the residual. Because propEG14_z was centered at zero, $a_{0i}$ represents the additive genetic value for AP at the mean propEG14 value in the analyzed population ($\mathrm{propEG}14_{z}=0$). The slope $a_{1i}$ represents the additive genetic change in AP associated with a one-standard-deviation increase in sustained heat-stress exposure.

The intercept and slope effects were assumed to follow: [a0a1] ~ N(0, ***K*** ⊗ $\;\Sigma_{a}$), where ***K*** was the pedigree-based relationship matrix ***A*** for the pedigree RNM or the combined pedigree–genomic relationship matrix H for the single-step genomic RNM. The intercept–slope genetic covariance matrix was $\Sigma_{a}$ = $\left[\begin{array}{cc} \sigma_{a_{0}}^{2} & \sigma_{a_{0}a_{1}}\\ \sigma_{a_{0}a_{1}} & \sigma_{a_{1}}^{2} \end{array}\right]$, where $\sigma_{a_{0}}^{2}$ represents the intercept genetic variance, $\sigma_{a_{1}}^{2}$ represents the slope genetic variance, and $\sigma_{a_{0}a_{1}}$ represents the genetic covariance between intercept and slope.

For the single-step genomic model, the inverse of ***H*** was constructed as follows [28,29]:

(7) $$H^{-1}=\;A^{-1}+\;\left[\begin{array}{cc} 0 & 0\\ 0 & G^{-1}-A_{22}^{-1} \end{array}\right],$$

where $A_{22}$ is the submatrix of A corresponding to genotyped animals and ***G*** is the genomic relationship matrix. The initial genomic relationship matrix $G_{0}$ was constructed using the method of VanRaden and blended with $A_{22}$ as $G=0.95G_{0}+0.05A_{22}$ [26,30,31]. The resulting matrix was subsequently tuned to the same scale as $A_{22}$ [32].

The genetic correlation between intercept and slope was calculated as $r_{g}$($\;a_{0},\;a_{1})=$ $\frac{\sigma_{a_{0}a_{1}}}{\sqrt{\sigma_{a_{0}}^{2}\;\times \;\sigma_{a_{1}}^{2}\;}}$. Residual variance was assumed to be homogeneous across the environmental gradient. All variance and covariance components were estimated using the average-information restricted maximum likelihood algorithm implemented in the BLUPF90 family programs (version 2.60) [33]. The significance of the random-slope genetic variance was evaluated using a one-tailed Wald test based on its estimate and standard error. Model fit was evaluated using the Akaike information criterion (AIC) [34]. Different starting values for the intercept–slope covariance matrix were tested to confirm convergence to a stable, non-boundary solution.

### 2.6. Environment-Specific Genetic Parameters and GEBV Re-Ranking

Environment-specific genetic parameters were derived from the estimated intercept–slope covariance matrix. For a given value (q) of propEG14_z, the additive genetic variance was calculated as

(8) $$V_{A}\left(q\right)=\sigma_{a_{0}}^{2}+2q\sigma_{a_{0}a_{1}}+q^{2}\sigma_{a_{1}}^{2},$$

where $\sigma_{a_{0}}^{2}$ is the intercept genetic variance, $\sigma_{a_{1}}^{2}$ is the slope genetic variance, and $\sigma_{a_{0}a_{1}}$ is the genetic covariance between the intercept and slope.

Environment-specific heritability was calculated as $h^{2}\left(q\right)=\frac{V_{A}\left(q\right)}{V_{A}\left(q\right)+\sigma_{e}^{2}}$, where $\sigma_{e}^{2}$ is the residual variance.

The genetic correlation between AP expressed at two environmental levels was calculated as $r_{g}\left(q_{1},q_{2}\right)=\frac{C_{A}\left(q_{1},q_{2}\right)}{\sqrt{V_{A}\left(q_{1}\right)V_{A}\left(q_{2}\right)}}$, where $q_{1}$ and $q_{2}$ are two values on the standardized propEG14_z gradient. Additive genetic variance and heritability were evaluated across the observed environmental range, and genetic correlations were calculated between pairs of environmental levels.

For gilt (i), the environment-specific GEBV obtained from the H-matrix RNM was calculated as follows [35]:

(9) $$\mathrm{GEB}V_{i}\left(q\right)=I_{i}+qS_{i},$$

where $I_{i}$ and $S_{i}$ are the predicted genetic intercept and slope, respectively. The intercept represents the predicted genetic value for AP at propEG14_z = 0, whereas the slope represents its change per one-standard-deviation increase in sustained heat-stress exposure.

### 2.7. Single-Step Genome-Wide Association Analysis

Single-step genome-wide association analyses were conducted separately for the genetic intercept and slope, estimated using the H-matrix RNM [36]. SNP effects for the two reaction-norm components were back-solved separately from the corresponding genomic breeding values using POSTGSF90 in the BLUPF90 family of programs (version 2.60) [37].

The genome was divided into non-overlapping windows of 20 consecutive SNPs. For each reaction-norm component, the genomic value attributable to a window was calculated by summing the genotype dosage multiplied by the estimated SNP effect across all SNPs within that window. The variance of these window-specific genomic values across genotyped animals was then expressed as a percentage of the total additive genetic variance of the corresponding reaction-norm component. Concordance between the intercept and slope was evaluated using Spearman’s rank correlation between their window-level variance contributions and the overlap between their top 20 windows.

### 2.8. QTL Overlap, Gene Annotation, and Functional Prioritization

Non-overlapping windows of 20 consecutive SNPs were ranked in descending order according to the proportion of additive genetic variance explained. The top 20 windows were retained for comparison and functional annotation, and no statistical *p*-value threshold was used for window selection. Reproductive QTL and association records aligned to Sscrofa11.1 were obtained from Pig QTLdb release 59 (https://bio.tools/shaziling_pigs, accessed on 30 September 2026). A window was considered to overlap a QTL when their genomic intervals shared at least one base pair.

Genes overlapping the selected windows were retrieved from Ensembl release 116 using the Sscrofa11.1 annotation (https://www.ensembl.org/, accessed on 15 August 2026). Cis-eQTL evidence for candidate genes in hypothalamus and blastocyst was obtained from PigGTEx (http://piggtex.farmgtex.org/, accessed on 15 August 2026).

## 3. Results

### 3.1. Phenotypic Description

Age at puberty (AP) was defined as the interval, in days, between the date of birth and the date of first observed estrus in gilts. After quality control, 4438 AP records from Yorkshire gilts were retained for analysis. The mean ± standard deviation of AP was 261.09 ± 22.95 days.

### 3.2. Selection of the Key Heat—Exposure Window

Using THI calculated from adjusted barn temperature and hourly relative humidity, 15 candidate heat-exposure scenarios were evaluated. Five exposure windows were defined relative to the date of first observed estrus: the preceding 7 days (−7, 0), 14 days (−14, 0), 30 days (−30, 0), 60 days (−60, 0), and 90 days (−90, 0). Each window was characterized using three environmental gradients: (1) the proportion of days with at least six consecutive hours of adjusted THI greater than 72 (prop-EG); (2) the mean daily minimum adjusted THI (mTHI-EG); (3) the cumulative heat load above the THI threshold of 72 (HLI).

AP was regressed separately on each candidate environmental gradient using an ordinary linear model that included farm and first-estrus year group as fixed effects. Model fit was compared using the Akaike information criterion (AIC), coefficient of determination (R^2^) and increase in R^2^ (ΔR^2^) relative to the baseline model. Among the 15 tested scenarios, propEG_14d showed the lowest AIC and the highest R^2^ and ΔR^2^, explaining an additional 9.22% of AP variation after accounting for farm and year-group effects (Table 1). The next-best model, propEG_7d, had a ΔAIC of 15.30, while the remaining models showed still larger ΔAIC values. Thus, propEG_14d provided the strongest statistical support among the candidate environmental gradients evaluated. Therefore, standardized propEG_14d, denoted as propEG14_z, was selected as the environmental gradient for subsequent analyses.

### 3.3. Sustained Heat-Stress Exposure Is Associated with Delayed AP and Genetically Heterogeneous Responses

After propEG14_z was selected as the most informative EG, we first evaluated whether it captured a systematic component of AP variation. In the baseline pedigree-based animal model without environmental covariates, the additive genetic variance and residual variance were 174.7 and 320.3, respectively, corresponding to a heritability estimate of 0.35. When propEG14_z was included as a fixed covariate, the AIC decreased from 39,198.12 to 38,790.02. The population-level fixed regression coefficient for propEG14_z was 7.64 days, indicating that a one-standard-deviation increase in sustained heat-stress exposure was associated with a 7.64-day increase in AP on average. Including propEG14_z reduced the estimated additive genetic variance from 174.7 to 129.9, whereas the residual variance decreased only slightly from 320.3 to 313.5. Accordingly, the heritability estimate decreased from 0.35 to 0.29 (Table 2). This pattern indicates that sustained heat-stress exposure before puberty captured an important systematic component of AP variation.

To investigate whether genetic differences existed in the response of AP to sustained heat-stress exposure, we subsequently fitted a pedigree-based RNM using the A matrix, in which propEG14_z was included as the environmental covariate for the animal-specific random slope. The estimated genetic variance of the random slope and its standard error were 294.91 and 16.81, respectively, and the variance was significantly greater than zero based on a one-tailed Wald test (*p* < 0.001) (Table 3). This result indicates that the response of AP to increasing sustained heat-stress exposure varied genetically among animals.

To evaluate whether this pattern was robust to the incorporation of genomic information, we further fitted the RNM using the H matrix, which combined pedigree and genomic relationships. The H-matrix model also detected a random-slope genetic variance significantly greater than zero (*p* < 0.001). The intercept–slope genetic covariances were −84.02 and −39.85 in the A- and H-matrix models, respectively, with corresponding genetic correlations of −0.430 and −0.524. Moreover, the H-matrix RNM had a lower AIC than the corresponding conventional H-matrix animal model (38,805.26 versus 39,302.50) (Table 3). Taken together, these results indicated that the model fit improved markedly when animal-specific genetic responses to sustained heat-stress exposure were accounted for.

### 3.4. Environment-Dependent Genetic Variation and Genetic Correlations Across Sustained Heat-Stress Exposure

**Figure 1:**
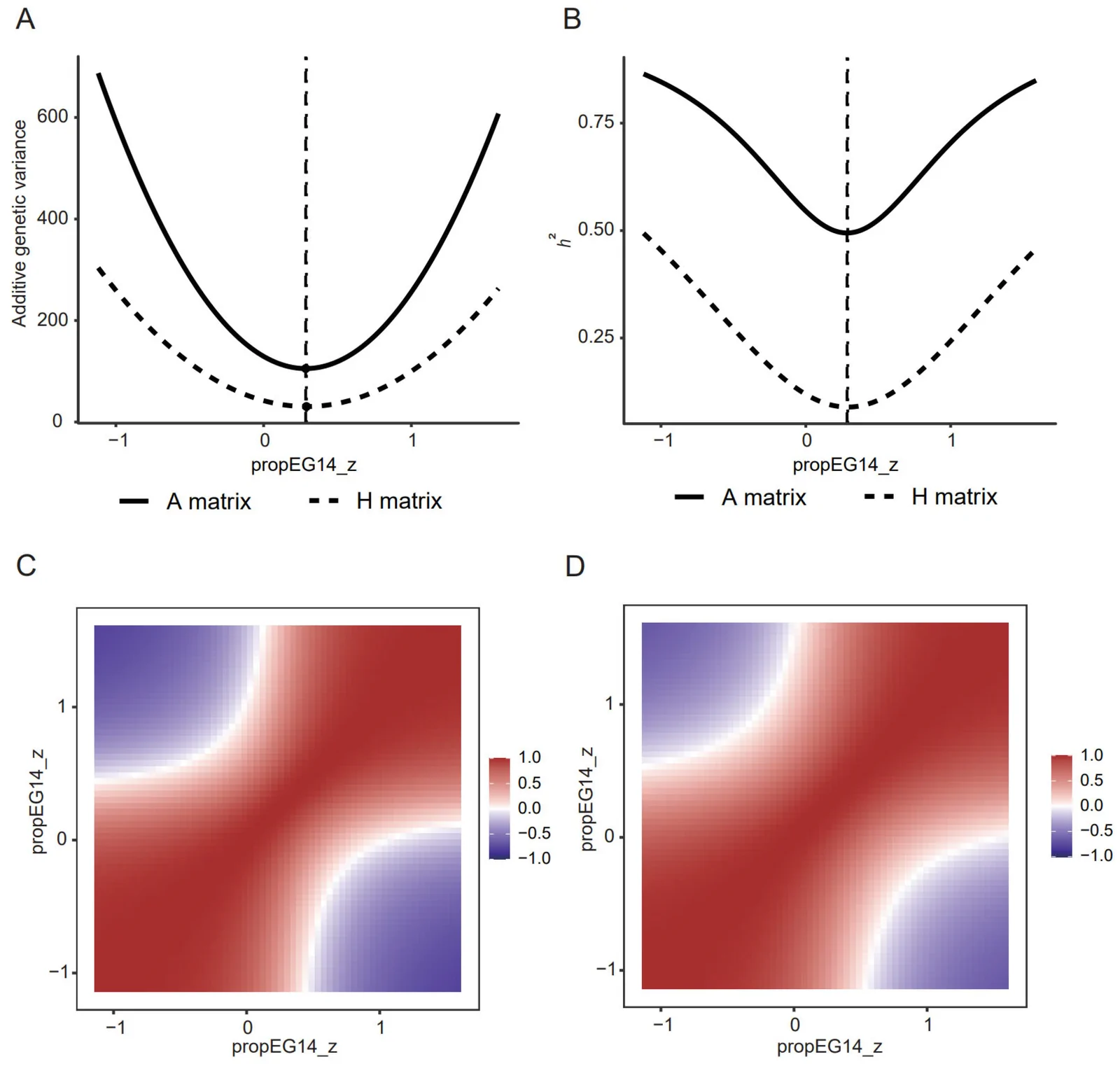
Environment-specific genetic parameters for age at puberty across the standardized sustained heat-stress gradient. (**A**) Additive genetic variance and (**B**) heritability estimated using the pedigree-based A-matrix and single-step genomic H-matrix reaction norm models. The dashed vertical lines in panels (**A**,**B**) indicate the propEG14_z values at which the H-matrix curves reached the minima. Pairwise genetic correlations between AP expressed at different levels of propEG14_z are shown for the (**C**) A-matrix and (**D**) H-matrix models.

Based on the H-matrix RNM, the environment-specific additive genetic variance of AP showed a nonlinear pattern across the sustained heat-stress gradient. The genetic variance initially decreased with increasing propEG14_z, reached its minimum at propEG14_z = 0.29, and subsequently increased as exposure increased further (Figure 1A). Environment-specific heritability showed a corresponding U-shaped pattern (Figure 1B). A similar trajectory was obtained from the A-matrix RNM, with the minimum genetic variance occurring at propEG14_z = 0.28. However, the absolute estimates differed markedly between the two models: the H-matrix RNM consistently produced lower additive genetic variance and heritability estimates than the A-matrix RNM, with the difference being most pronounced around the intermediate exposure level. Despite these differences in magnitude, both models showed nearly identical U-shaped trajectories and turning points. This pattern indicates that genetic differences in AP were smallest around intermediate heat exposure and became more pronounced toward both ends of the environmental gradient, while the consistency between the two models suggests that this environment-dependent pattern was not driven solely by the choice of relationship matrix.

Genetic correlations between AP expressed at different levels of propEG14_z were highest between adjacent environments and progressively declined as the distance between environmental levels increased (Figure 1C,D). Correlations became negative between the most contrasting low- and high-exposure environments, indicating substantial changes in the genetic expression of AP across the sustained heat-stress gradient. Both the A- and H-matrix RNMs produced similar genetic-correlation patterns, indicating that the inferred genotype-by-environment interaction was robust to the choice of relationship matrix.

### 3.5. Re-Ranking of Gilts Across Levels of Sustained Heat-Stress Environments

**Figure 2:**
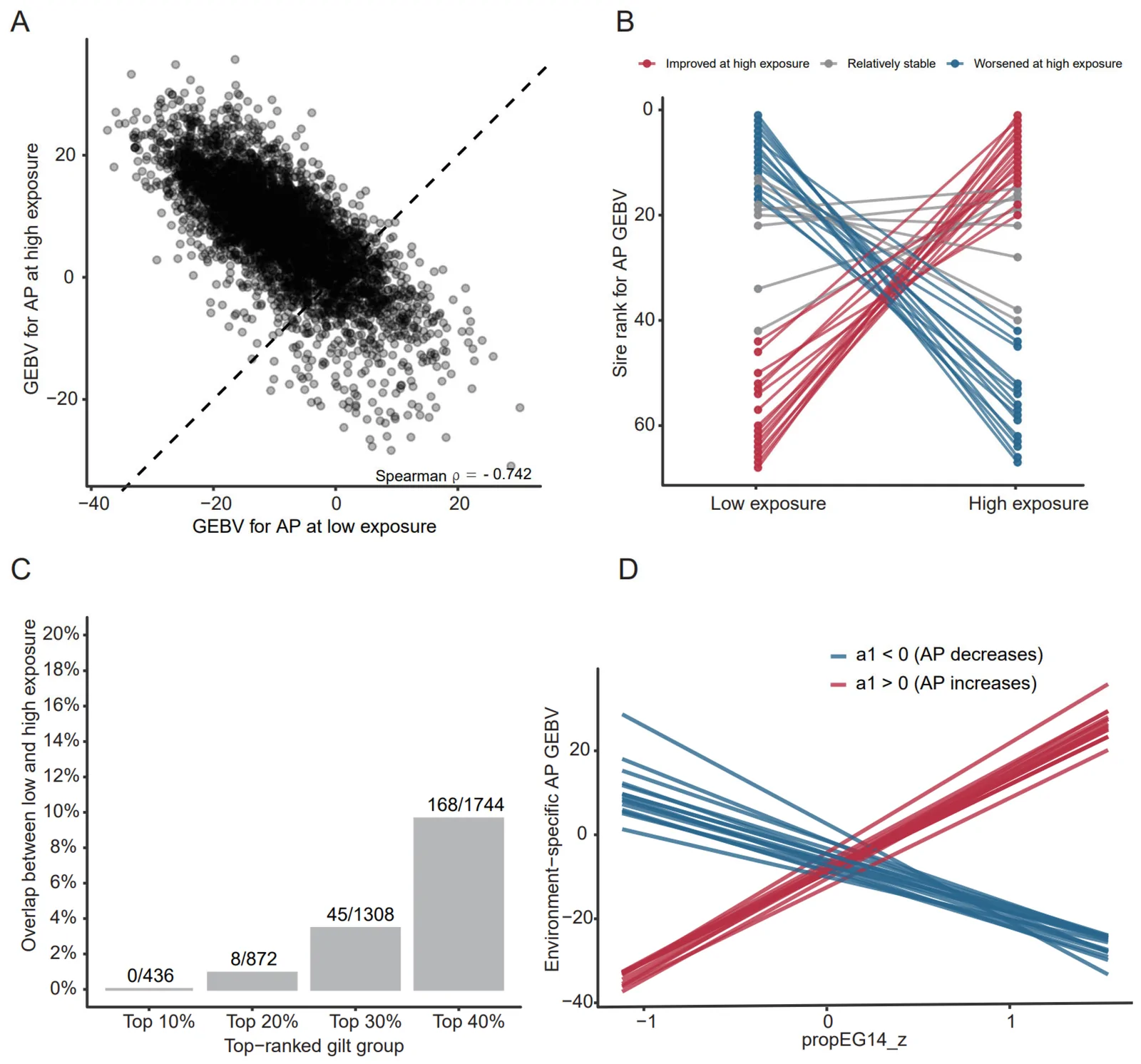
Re-ranking of gilts across low and high levels of sustained heat-stress exposure based on the H-matrix reaction norm model. (**A**) Relationship between environment-specific GEBVs for AP predicted at the 10th and 90th percentiles of propEG14_z. The dashed diagonal line represents the identity line (y = x). (**B**) Comparison of gilt rankings between the two exposure levels. (**C**) Numbers and percentages of gilts shared between the most favorable 10%, 20%, 30%, and 40% groups. (**D**) Genetic reaction norms of gilts ranked among the 15 most favorable animals at either exposure level. Lower GEBVs indicate more favorable genetic merit for earlier puberty.

For each gilt included in the H-based reaction norm analysis, environment-specific GEBVs for AP were predicted at two predefined levels of the environmental gradient: the 10th percentile of propEG14_z, representing low sustained heat-stress exposure, and the 90th percentile, representing high exposure. The Spearman rank correlation between GEBVs predicted at these two environmental levels was −0.742, indicating pronounced re-ranking of gilts across the sustained heat-stress gradient (Figure 2A). Because lower AP GEBVs represent genetically earlier puberty, gilts ranked favorably at the low-exposure level frequently became less favorable at the high-exposure level, and vice versa (Figure 2B).

The overlap between favorable gilt groups was extremely limited. None of the top 10% of gilts under low-exposure conditions remained within the top 10% under high-exposure conditions. Only 8 of 872 gilts were shared between the two top-20% groups, corresponding to an overlap of 0.92%. The overlaps increased to 45 of 1308 gilts for the top 30% and 168 of 1744 gilts for the top 40%, but remained low at 3.44% and 9.63%, respectively (Figure 2C).

To further illustrate the re-ranking of gilts, reaction norms were plotted for gilts ranked within the top 15 under either low- or high-exposure conditions (Figure 2D). Gilts with positive genetic slopes showed increasing AP GEBVs as propEG14_z increased, resulting in less favorable genetic merit for earlier puberty at higher exposure levels. Conversely, gilts with negative slopes showed decreasing AP GEBVs and became relatively more favorable as exposure increased. The individual reaction norms crossed extensively across the environmental gradient. A comparable pattern of environment-dependent re-ranking was obtained from the pedigree-based RNM, supporting the robustness of the results to the relationship matrix used (Figure S1).

### 3.6. Shared Genomic Architecture Underlying AP and Its Response to Sustained Heat-Stress Exposure

**Figure 3:**
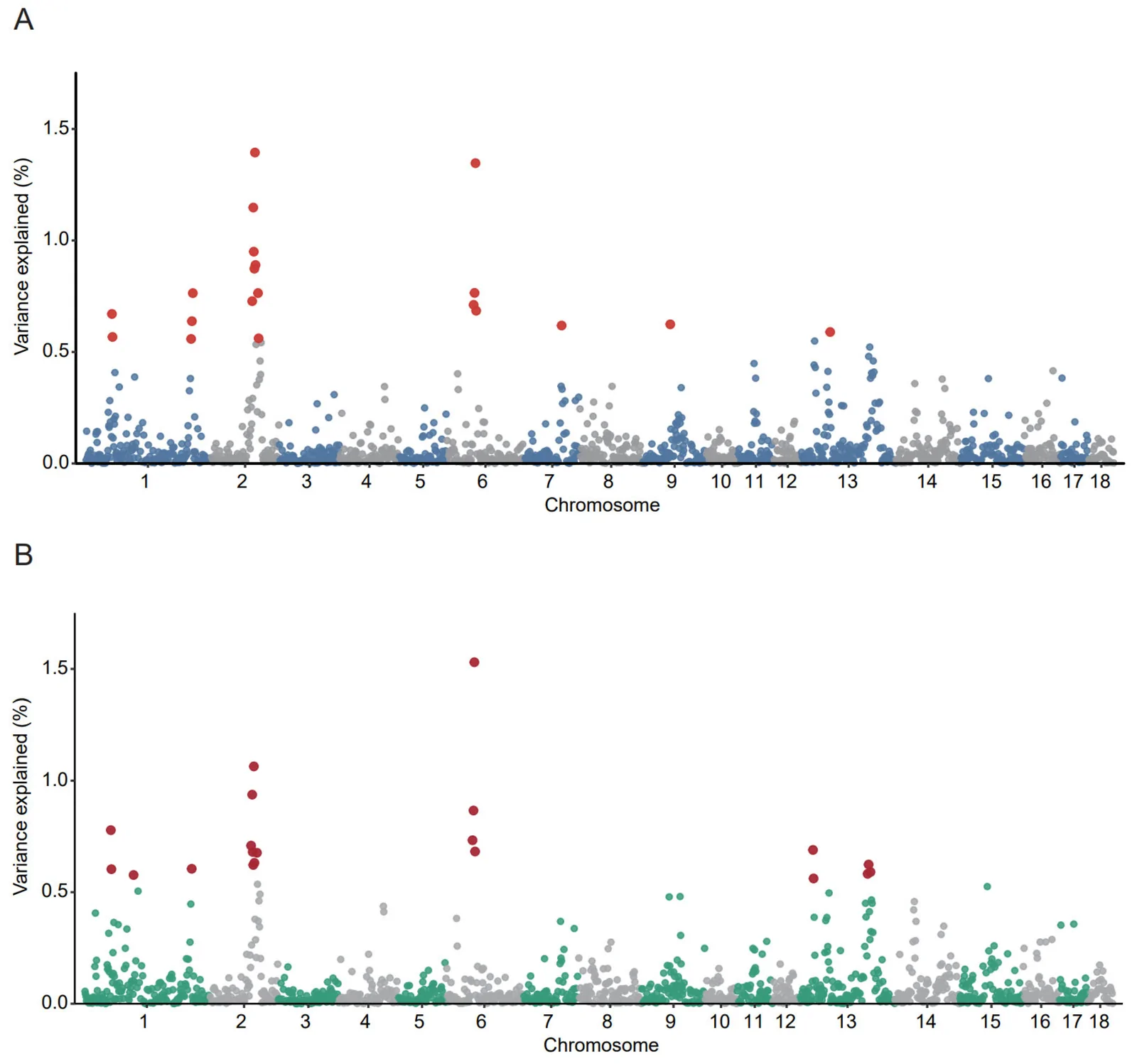
Genome-wide distribution of window-level genetic variance contributions for the intercept and slope of the age-at-puberty reaction norm. The proportions of genetic variance explained by non-overlapping 20-SNP windows are shown for the (**A**) intercept and (**B**) slope estimated using the H-matrix reaction norm model. Each point represents one genomic window, and chromosomes are distinguished by alternating colors. Red points indicate the 20 windows with the largest variance contributions for the corresponding component, whereas non-highlighted windows are shown in alternating colors to distinguish adjacent chromosomes.

Single-step genome-wide association analyses were performed for the random intercept and random slope estimated from the H-based RNM to characterize the genomic architecture underlying AP and its genetic response to sustained heat-stress exposure. The random intercept represented genetic value for AP at the mean level of the standardized environmental gradient (propEG14_z = 0), whereas the random slope represented the animal-specific genetic response to increasing sustained heat-stress exposure. Genomic variance was summarized using non-overlapping windows of 20 consecutive SNPs. Windows were ranked according to the proportion of additive genetic variance explained. Genetic variance for both components was distributed across the genome, with the largest window-level contributions observed on SSC2 and SSC6 (Figure 3A,B). For the random intercept, the window on SSC2 at 97.30–98.40 Mb explained the greatest proportion of genetic variance (1.394%), followed by SSC6 at 62.42–64.31 Mb (1.347%) and SSC2 at 93.46–94.86 Mb (1.148%). For the random slope, the largest contribution was observed for the SSC6 window at 62.42–64.31 Mb (1.530%), followed by SSC2 at 97.30–98.40 Mb (1.064%) and SSC2 at 93.46–94.86 Mb (0.937%) (Table S1).

**Figure 4:**
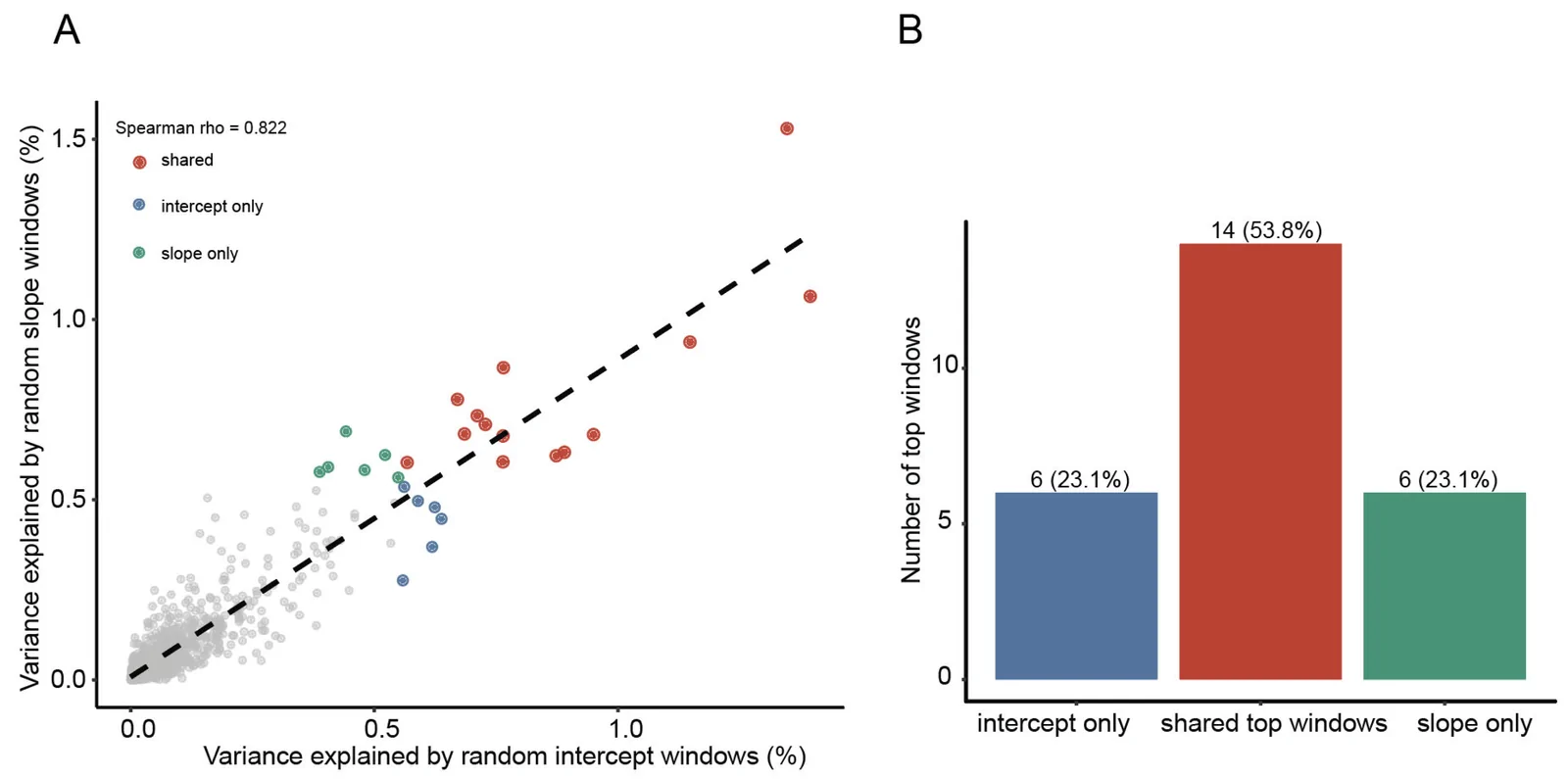
Concordance and overlap between genomic window contributions to the intercept and slope of the age-at-puberty reaction norm. (**A**) Relationship between the proportions of genetic variance explained by corresponding 20-SNP windows for the intercept and slope. Red points indicate windows included in both top-20 lists, blue points indicate windows included only in the intercept top 20, green points indicate windows included only in the slope top 20, and gray points indicate all remaining windows. The dashed line represents the identity line (y = x). (**B**) Numbers of windows unique to the intercept top 20, shared between the two top-20 lists, and unique to the slope top 20.

Although the relative contributions of individual windows differed between the two components, their genome-wide patterns showed strong concordance. Across corresponding genomic windows, the proportions of genetic variance explained by the intercept and slope were strongly rank-correlated (Spearman’s ρ = 0.822; Figure 4A), indicating that regions contributing substantially to genetic variation in AP at the mean environmental level also tended to contribute to variation in the genetic response to sustained heat-stress exposure. This concordance was also evident among the leading genomic windows, with 14 of the top 20 windows ranked by the proportion of genetic variance explained for the intercept also ranking among the top 20 for the slope. The remaining six windows in each set were component-specific (Figure 4B) (Table S1).

### 3.7. Integrative Functional Annotation Prioritizes Candidate Genes for AP and Heat-Stress Responsiveness

To assess the biological relevance of the leading genomic regions, the 26 nonredundant windows represented among the top 20 windows for either the random intercept or random slope were integrated with previously reported reproductive QTLs, and gene annotations. These regions comprised 14 shared, six intercept-specific, and six slope-specific windows (Figure 4B). Of the 26 windows, 20 overlapped 67 reproductive QTL records (Table S2). The overlapping QTL included traits related to age at puberty, litter size and related offspring traits, teat number, gestation length, semen-quality traits, and piglet mortality. Notably, previously reported AP QTL overlapped two shared windows on SSC2 at 94.87–95.65 and 95.67–97.29 Mb, as well as an intercept-specific window on SSC9 at 59.92–60.79 Mb. Across all 26 windows, 660 annotated genes were identified, of which 420 were protein-coding genes, providing the candidate set for subsequent functional prioritization (Table S3).

Among the shared regions, the SSC2 window at 94.87–95.65 Mb was particularly notable because it overlapped a previously reported AP QTL and contained *CCNH*. The Pig Genotype-Tissue Expression (PigGTEx) resource reported significant cis-eQTL associations for *CCNH* in both hypothalamus and blastocyst (Table S4), supporting its prioritization as a candidate gene within this region. Previous functional studies have also demonstrated that CCNH is a component of the CDK-activating kinase complex and is required for meiotic progression in porcine oocytes [38]. These independent lines of evidence make CCNH a biologically plausible candidate for further investigation.

The intercept-specific regions contained several candidate genes with previously reported functions relevant to reproductive maturation, among which *TLE4* and *PSAT1* were particularly notable. *TLE4* encodes a transcriptional corepressor of the Groucho/TLE family and has been implicated in the transcriptional regulation of *gonadotropin-releasing hormone* (*GnRH*) [39,40]. PSAT1, a key enzyme in de novo serine biosynthesis, has been associated with cumulus-cell expression patterns related to oocyte nuclear maturation and embryo developmental competence [41]. These previously reported functions support the biological plausibility of *TLE4* and *PSAT1* as candidates within the intercept-specific regions, particularly in relation to neuroendocrine regulation and metabolic processes associated with oocyte maturation.

The slope-specific regions contained two groups of candidate genes with reported functions potentially relevant to heat responsiveness. The first group comprised *FECH*, *DAPK2*, and *SETD2*, for which previous studies have reported functions related to mitochondrial activity, redox regulation, and cellular stress homeostasis [42,43]. The second group comprised genes with reported functions related to reproductive and developmental processes. *DPPA2* and *DPPA4* have been implicated in transcriptional and epigenetic programs during early development, *PTHLH*-*PTH1R* signaling has been associated with follicular-cell proliferation and steroidogenic activity, *RBPMS2* contributes to female gonadal fate and oocyte differentiation, and *OAZ2* regulates intracellular polyamine homeostasis [44,45]. Together, these candidates suggest that slope-specific genomic variation may contribute to variation in AP responsiveness through both cellular stress-adaptation mechanisms and the environmental sensitivity of reproductive-developmental processes.

## 4. Discussion

This study showed that sustained heat-stress exposure before puberty was associated with delayed AP and revealed substantial genetic heterogeneity in this response. Both pedigree- and genomic-based RNM detected genetic variation in environmental sensitivity, while changes in genetic variance and correlations across the exposure gradient resulted in pronounced re-ranking of gilts. Genomic analyses identified substantial overlap between regions contributing to AP at mean exposure (intercept) and those contributing to sensitivity to increasing exposure (slope), together with component-specific regions. Functional annotation further implicated neuroendocrine regulation, oocyte and follicular development, mitochondrial and redox homeostasis, and cellular stress responses. Collectively, these findings extend previous evidence for adverse effects of heat stress on gilt reproductive development by demonstrating that sensitivity of AP to sustained heat exposure is itself genetically heterogeneous and potentially amenable to selection.

### 4.1. Sustained Heat-Stress Exposure Before Puberty Is Associated with Delayed AP

The present study identified a clear association between sustained heat-stress exposure and delayed puberty in gilts. This finding is consistent with earlier experimental and field evidence of delayed reproductive maturation under elevated thermal exposure in gilts [9,14]. The adverse reproductive consequences of heat exposure are not restricted to pubertal timing. In commercial pig populations, elevated temperatures during specific periods around insemination were associated with reduced farrowing rate and number of total born piglets, with stronger relationships observed in gilts than in multiparous sows [41]. Genomic reaction-norm analyses of more than 80,000 records from four maternal pig lines further identified environment-dependent variation in number of total born piglets, number born alive, and average piglet birth weight, together with heritable differences in heat sensitivity [19]. Comparable effects have been reported in dairy cattle: heat exposure before and after breeding was associated with a marked reduction in conception rate, whereas elevated THI during the peri-implantation period increased the risk of subsequent early fetal loss [46]. Experimental evidence further showed that maternal heat stress during late gestation compromised calf survival and subsequent performance [47]. Collectively, these studies indicate that thermal stress can impair reproductive and offspring output across species and reproductive stages.

### 4.2. Genetic Heterogeneity and Environment-Dependent Re-Ranking

The significant random-slope variance detected using both the A- and H-matrix reaction norm models indicates that the association between sustained heat exposure and AP was not uniform across gilts. Whereas the population-level regression estimated in the preceding analysis describes the average increase in AP with increasing exposure, the random slope represents genetic differences among gilts in the magnitude and direction of this response. The marked improvement in model fit after including animal-specific slopes further supports the presence of biologically relevant genotype-by-environment interactions. Heritable variation in heat sensitivity has previously been reported for litter size, piglet birth weight, farrowing interval, and other reproductive traits in maternal pig populations [19,20,21]. The negative genetic correlation between the intercept and slope (−0.524) provides further insight into the structure of this variation. Because a lower AP is favorable, this correlation suggests that gilts genetically predisposed to earlier puberty at the mean exposure level tended, on average, to show greater increases in AP as heat exposure intensified. This pattern is consistent with a potential antagonistic relationship between genetic merit for earlier puberty and environmental stability. Considerable variation remained around this overall tendency, indicating that earlier puberty and lower sensitivity to increasing heat exposure are not genetically incompatible. The exposure-dependent changes in genetic variance and the declining genetic correlations between increasingly contrasting environments further indicate that the genetic expression of AP changes across the sustained heat-stress gradient.

Reaction norm models enable the prediction of environment-specific GEBVs across different levels of sustained heat-stress exposure [20,26]. In the present study, the Spearman correlation between GEBVs predicted at the 10th and 90th percentiles of propEG14_z was −0.742, and none of the most favorable 10% of gilts under low exposure remained within the most favorable 10% under high exposure. The moderate negative genetic correlation between intercept and slope (r_g = −0.524) suggests that genotypes associated with earlier puberty under average thermal exposure tended, on average, to show greater increases in AP as heat exposure intensified. One possible biological explanation is that pubertal attainment depends on coordinated metabolic and neuroendocrine signals supporting activation of the hypothalamic–pituitary–gonadal axis. Heat stress can alter energy metabolism and stress signaling and suppress gonadotropin secretion and follicular development in pigs [6,48]. Thus, genotypes that express an earlier-puberty phenotype under favorable conditions may lose part of this advantage when thermal stress disrupts the metabolic and endocrine processes supporting reproductive maturation. The extensive crossing of individual reaction norms therefore reflects not only differences in environmental sensitivity but also a substantial reversal of relative genetic merit. Consequently, selection based solely on AP at the mean exposure level may fail to identify gilts that remain favorable under sustained heat stress. Selection based on slope alone would also be insufficient because a flat or negative slope does not guarantee a favorable AP across the relevant environmental range. A more appropriate strategy would be to select on predicted AP in the target production environment or to construct an index that jointly considers AP under average exposure and sensitivity to increasing heat load. Gilts combining genetically early puberty with limited deterioration across the environmental gradient would represent desirable candidates for reproductive robustness. Genomic reaction norm evaluation may facilitate such selection, although the predictive accuracy and stability of environment-specific GEBVs should be validated in independent populations before implementation.

### 4.3. Genomic Links Between Reproductive Maturation and Heat Responsiveness

The strong rank correlation between the proportions of genetic variance explained by corresponding intercept and slope windows (Spearman’s ρ = 0.822), together with the overlap of 14 of their top 20 windows, indicates that genomic regions contributing to genetic variation in AP at the mean exposure level also tended to contribute to its sensitivity to increasing sustained heat exposure. However, this concordance should be interpreted as overlap in regional genomic contributions. Because the ssGWAS results were summarized as proportions of genetic variance explained by 20-SNP windows, they do not indicate whether alleles within these regions affected the intercept and slope in the same direction. Nor does window overlap demonstrate the presence of a shared causal variant, because it may reflect either pleiotropic variants or distinct variants in linkage disequilibrium. Thus, the results support a substantially shared genomic basis at the regional level, while the component-specific windows indicate additional genetic influences that differentiate AP at the mean exposure level from its heat responsiveness.

Functional evidence within the shared and intercept-specific regions connects this genomic pattern with pathways involved in reproductive maturation. The shared SSC2 window at 94.87–95.65 Mb provided the strongest convergence of positional, regulatory, and functional evidence because it overlapped a previously reported AP QTL and contained *CCNH*, whose expression was associated with cis-eQTLs in both hypothalamus and blastocyst. Experimental manipulation of porcine oocytes has demonstrated that the CDK7–CCNH complex is required for normal meiotic progression [38]. Among the intercept-specific candidates, TLE4 belongs to the Groucho/TLE family of transcriptional corepressors, members of which regulate *GnRH* transcription through interactions with MSX1 and OCT1 [39,40]. PSAT1, a key enzyme in de novo serine biosynthesis, has also been associated with oocyte nuclear maturation through its expression in human cumulus cells [46]. Collectively, these candidates suggest that genetic differences in AP at the mean exposure level may involve central neuroendocrine regulation, oocyte meiotic maturation, and the metabolic support provided by surrounding somatic cells. Nevertheless, these data prioritize plausible candidates rather than establish causal genes, because the eQTL and ssGWAS signals have not been formally colocalized or functionally validated.

The slope-specific regions point to two complementary mechanisms that may underlie genetic differences in AP responsiveness. The first, represented by *FECH*, *DAPK2*, and *SETD2*, involves mitochondrial function, redox regulation, and cellular stress homeostasis. Experimental depletion of *DAPK2* impaired mitochondrial function and increased cellular oxidative stress [42], whereas loss of *SETD2* increased reactive oxygen species through the downregulation of antioxidant genes [43]. These functions are consistent with experimental evidence that seven days of cyclic heat stress altered ovarian proteins involved in metabolism, protein processing, immune activation, and gene regulation in prepubertal gilts, including changes observed relative to pair-fed controls [16]. The second mechanism involves the environmental sensitivity of reproductive-developmental processes. *PTHLH*, which signals through *PTH1R*, has been shown to stimulate follicular-cell proliferation and steroidogenesis during follicle selection in hens [44], whereas *RBPMS2* is required for oocyte progression and ovarian fate in zebrafish [45]. Other slope-specific candidates, including *DPPA2*, *DPPA4*, and *OAZ2*, further broaden this signal toward germ-cell and follicular-developmental regulation. Together, these findings suggest that genetic differences in AP responsiveness may arise from the interaction between general cellular stress-management capacity and the sensitivity of ovarian and germ-cell developmental processes to sustained heat exposure. However, most supporting functional evidence was obtained from other species or nonreproductive cellular systems; these genes should therefore be regarded as candidates for heat responsiveness rather than confirmed porcine heat-tolerance genes.

### 4.4. Implications, Limitations, and Future Directions

The negative rank correlation between GEBVs predicted at the 10th and 90th percentiles of the exposure gradient, together with the absence of overlap between the most favorable 10% of gilts, demonstrates that the thermal conditions of the target production system should be explicitly considered when selecting for AP. In hot and humid regions, priority should be given to gilts that maintain favorable predicted AP under high heat exposure and show limited deterioration as exposure increases. Using such gilts as parents may help improve reproductive heat robustness in subsequent generations.

Several limitations should be considered when interpreting the present results. First, gilts were not compared across thermal conditions during a unified prepubertal age interval. Because the exposure windows were defined relative to each gilt’s date of first observed estrus, the selected 14-day window occurred at different ages among animals. Therefore, the present result identifies heat exposure immediately preceding first observed estrus as the environmental descriptor most strongly associated with AP, but it does not identify a fixed chronological-age window during which all gilts are maximally susceptible to heat. Establishing such an age-specific sensitive period would require comparisons across common prepubertal age intervals or controlled heat exposure of gilts at comparable developmental stages. Second, the analysis involved two commercial Yorkshire populations. The estimated covariance structure, environmental sensitivity, and environment-specific rankings may differ among genetic lines, farms, and climatic regions. External validation in other major maternal breeds, particularly Landrace, would therefore be valuable for determining whether the G × E patterns identified here are transferable across maternal genetic backgrounds. More broadly, the stability and predictive accuracy of environment-specific GEBVs require validation in independent populations across breeds, farms, generations, and climatic regions before they can be reliably implemented in practical breeding programs. Third, individual body weight and body-condition measurements were not consistently available, preventing evaluation of whether variation in growth or body condition contributed to differences in AP or its association with heat exposure. Finally, the approximately 28,000 markers and non-overlapping 20-SNP windows provided regional rather than variant-level resolution. Because the physical size represented by a 20-SNP window depends on local marker density and linkage disequilibrium, an associated window may span a relatively broad genomic interval containing multiple linked variants and genes. Consequently, the ssGWAS results should be interpreted as identifying candidate genomic regions rather than pinpointing individual causal variants or genes. The overlap with previously reported QTL and cis-eQTL information increased the biological plausibility of genes such as CCNH and other prioritized candidates, but these overlaps do not demonstrate that the nominated gene, or the same underlying causal variant, is responsible for the observed association. Higher-density genotypes or sequence-level data, followed by fine-mapping and formal colocalization analyses, will be required to resolve these regions more precisely.

Future studies should complement the validated environmental gradient used here with continuously recorded within-barn temperature and humidity and individual-level indicators such as body temperature, feed intake, respiration rate, and activity. Combining these measurements would help distinguish ambient thermal exposure from variation in individual biological responses. At the genomic level, high-density genomic information, formal colocalization, and fine-mapping should be integrated with transcriptomic profiling of hypothalamic, ovarian, and uterine tissues collected under controlled heat exposure. This integrated design would test whether shared intercept–slope windows reflect common causal signals and whether component-specific candidates show heat-responsive regulation in reproductively relevant tissues.

## 5. Conclusions

Sustained heat-stress exposure immediately preceding puberty was associated with delayed AP, and significant random-slope genetic variance demonstrated genetic heterogeneity in AP responsiveness. The negative genetic correlation between the intercept and slope suggested a tendency for genetic merit for earlier puberty at the mean exposure level to be associated with greater sensitivity to increasing heat load. Pronounced re-ranking of GEBVs across the environmental gradient further demonstrated that the relative genetic merit of gilts can depend strongly on thermal conditions. The shared yet partially distinct genomic-window architecture further indicated that baseline AP and heat responsiveness are genetically connected but not identical. These findings support the use of AP responsiveness to heat exposure as a reproductive-resilience phenotype and provide a basis for environment-specific or robustness-oriented genomic evaluation. However, the stability and predictive accuracy of environment-specific GEBVs require validation in independent populations, breeds, farms, generations, and climatic regions before reliable implementation in practical breeding programs. Future studies integrating more direct measurements of thermal exposure and physiological responses with higher-resolution genomic and functional data will be important for validating the biological mechanisms underlying AP sensitivity to heat.

## Figures and Tables

**Table 1:** Comparison of 15 candidate heat-related environmental gradients across five exposure windows preceding first observed estrus.

Environmental Gradient	Period ^a^	Beta	*p*-Value	R^2 b^	ΔR^2 c^	AIC	ΔAIC ^d^
propEG_14d	(−14, 0)	20.96	1.27 × 10^−99^	0.1764	0.0922	39,011.8	0.00
propEG_7d	(−7, 0)	20.43	2.67 × 10^−96^	0.1735	0.0893	39,027.1	15.30
HLI_14d	(−14, 0)	0.01858	3.52 × 10^−93^	0.1708	0.0866	39,041.5	29.65
propEG_30d	(−30, 0)	20.19	2.12 × 10^−89^	0.1675	0.0833	39,058.9	47.05
HLI_7d	(−7, 0)	0.03589	1.23 × 10^−86^	0.1650	0.0809	39,071.6	59.76
HLI_30d	(−30, 0)	0.008523	2.53 × 10^−85^	0.1639	0.0797	39,077.6	65.80
HLI_60d	(−60, 0)	0.003912	1.01 × 10^−60^	0.1420	0.0578	39,190.7	178.91
propEG_60d	(−60, 0)	17.38	4.71 × 10^−58^	0.1395	0.0554	39,203.0	191.18
mTHIEG_14d	(−14, 0)	0.8911	1.33 × 10^−54^	0.1364	0.0522	39,218.9	207.03
mTHIEG_7d	(−7, 0)	0.8315	2.17 × 10^−53^	0.1353	0.0511	39,224.4	212.59
mTHIEG_30d	(−30, 0)	0.8827	5.91 × 10^−45^	0.1276	0.0434	39,263.2	251.32
propEG_90d	(−90, 0)	12.44	1.20 × 10^−23^	0.1079	0.0237	39,360.8	348.93
HLI_90d	(−90, 0)	0.001854	2.24 × 10^−23^	0.1077	0.0235	39,362.0	350.16
mTHIEG_60d	(−60, 0)	0.6452	4.93 × 10^−19^	0.1036	0.0194	39,381.8	369.97
mTHIEG_90d	(−90, 0)	0.2794	0.0004705	0.0897	0.0055	39,449.1	437.30

^a^ Exposure windows were defined relative to the date of first observed estrus; negative values indicate days preceding the first observed estrus. ^b^ R^2^ represents the coefficient of determination of the full model. ^c^ ΔR^2^ represents the increase in R^2^ compared with the baseline model including only farm and first-estrus year group. ^d^ ΔAIC was calculated relative to the best-fitting model.

**Table 2:** Variance estimates from pedigree-based animal models fitted without and with propEG14_z as a fixed covariate.

Model	β of EG ^a^	$\sigma_{a}^{2}$	$\sigma_{e}^{2}$	h^2^
Baseline animal model	—	174.7	320.3	0.35
Animal model with propEG14_z	7.64	129.9	313.5	0.29

^a^ β of EG is the change in AP, in days, associated with a one-standard-deviation increase in propEG14.

**Table 3:** Genetic (co)variance estimates from pedigree-based and single-step genomic reaction norm models for age at puberty.

Relationship Matrix	$\sigma_{a0}^{2}$	$\sigma_{a0a1}$	$\sigma_{a1}^{2}$	SE	$\sigma_{e}^{2}$	z	One-Tailed *p*	$r_{a0,a1}$
A	129.31	−84.02	294.91	16.81	107.7	17.543	3.38 × 10^−69^	−0.430
H	42.11	−39.85	137.52	13.77	311.48	9.985	8.89 × 10^−24^	−0.524

## Data Availability

The datasets analyzed during this study are available from the authors upon reasonable request.

## Supplementary Materials

The following supporting information can be downloaded at https://www.mdpi.com/article/10.3390/ani16193085/s1. Figure S1: Re-ranking of gilts across low and high levels of sustained heat-stress exposure based on the pedigree-based A-matrix reaction norm model. (A) Relationship between environment-specific EBVs for AP predicted at the 10th and 90th percentiles of propEG14_z. (B) Comparison of gilt rankings between the two exposure levels. Gilts whose rankings improved or worsened by at least 30 positions were classified accordingly, whereas changes of fewer than 30 positions were classified as relatively stable. (C) Numbers and percentages of gilts shared between the most favorable 10%, 20%, 30%, and 40% groups at the two exposure levels. (D) Genetic reaction norms for the union of the 15 most favorable gilts at the low- and high-exposure levels. Dashed vertical lines indicate the 10th and 90th percentiles of propEG14_z. Lower EBVs indicate more favorable genetic merit for earlier puberty; Table S1: Genomic windows ranked among the Top 20 for the intercept or slope of the age-at-puberty reaction norm; Table S2: Reproductive QTL records from Pig QTLdb overlapping the 26 leading genomic windows identified for the intercept or slope; Table S3: Genes annotated within the 26 leading genomic windows identified for the intercept or slope; Table S4: Reported cis-expression quantitative trait locus associations for CCNH in hypothalamus and blastocyst obtained from PigGTEx.

## Abbreviations

The following abbreviations are used in this manuscript:

AIC	Akaike information criterion
AP	Age at puberty
EBV	Estimated breeding value
ECMWF	European Centre for Medium-Range Weather Forecasts
EG	Environmental gradient
eQTL	Expression quantitative trait locus
GEBV	Genomic estimated breeding value
G × E	Genotype-by-environment interaction
HLI	Cumulative heat load above the THI threshold of 72
IBS	Identity by state
MAF	Minor allele frequency
mTHI-EG	Environmental gradient based on mean daily minimum adjusted THI
NASA	National Aeronautics and Space Administration
prop-EG	Environmental gradient based on the proportion of sustained heat-stress days
QTL	Quantitative trait locus
RH	Relative humidity
RNM	Reaction norm model
SD	Standard deviation
SNP	Single-nucleotide polymorphism
SSC	Sus scrofa chromosome
ssGWAS	Single-step genome-wide association analysis
THI	Temperature–humidity index
