# Supplementary material for: Single-Step Genomic Reaction Norm and ssGWAS Reveal Heat-Load-Dependent Genetic Architecture of Age at Puberty in Yorkshire Gilts
Source: Animals (Basel). 2026 Sep 30;16(19):3085. doi: 10.3390/ani16193085 (PMC13663369; doi:10.3390/ani16193085)
Supplement: Supplementary file 1 [file animals-16-03085-s001.zip › Supplementary Figure.docx]

**Figure S1.** Re-ranking of gilts across low and high levels of sustained heat-stress exposure based on the pedigree-based A-matrix reaction norm model. (A) Relationship between environment-specific EBVs for AP predicted at the 10th and 90th percentiles of propEG14_z. (B) Comparison of gilt rankings between the two exposure levels. Gilts whose rankings improved or worsened by at least 30 positions were classified accordingly, whereas changes of fewer than 30 positions were classified as relatively stable. (C) Numbers and percentages of gilts shared between the most favorable 10%, 20%, 30%, and 40% groups at the two exposure levels. (D) Genetic reaction norms for the union of the 15 most favorable gilts at the low- and high-exposure levels. Dashed vertical lines indicate the 10th and 90th percentiles of propEG14_z. Lower EBVs indicate more favorable genetic merit for earlier puberty.
